# Editorial

**DOI:** 10.4314/ahs.v22i2.1

**Published:** 2022-06

**Authors:** James K Tumwine

*Editor's choice:* You are very welcome to this June 2022 issue of *African Health Sciences* in which the authors have contributed high quality papers relevant to health in Africa and the diaspora. This issue has four main themes: infections (1–25); *non communicable diseases* (26–44); *sexual reproductive health +child health* (45–66); and *others* 67–82. Clearly quite a diverse and large collection of scientific papers. Why large? Well, because we have had a backlog, largely due to the Covid-19 pandemic. Here we look at some of the themes in this issue.

*Infections:* We have 25 papers in this section including *Moringa* adjunct therapy to HAART^1^; loss to follow up among HIV infected people^2^; HIV and colorectal cancer^3^; HIV self-testing in Kenya and Tanzania^4^,^5^; and adherence to HIV^6^. The infectious disease theme continues with papers on Covid-19 in several African countries on very specific aspects^7^–^9^. Then we have articles on human papilloma virus^10^–^11^; hepatis B^12^ and acute flaccid paralysis^13^. Others are on *Helicobacter pylori*^14^–^15^; beta lactamase^16^; tuberculosis^17^–^20^ and malaria^21^–^23^. We end this section with papers on nematodes^24^ and hydatidosis^25^.

*Non communicable diseases:* This theme is quite diverse. It includes: cardiovascular disease^26^–^31^; cancer^32^–^38^. The rest include: kidney disease^39^–^40^; foreign bodies in the esophagus and liver cirrhosis^41^–^42^; and GI endoscopy^43^–^44^.

*Sexual reproductive health +child health* (45–66); This subtheme includes articles on menstruation^45^, male sex hormones^46^, erectile dysfunction^47^; use of aphrodisiacs^48^ intergenerational sexual relationships^49^; and thyroid function and infertility^50^. It also includes papers on teenage pregnancy^51^–^52^ and adolescent child bearing^53^. We then have articles on pre-eclampsia^54^; child birth^55^; maternal mortality^56^–^57^; and caesarean section^58^. The child group includes papers on birth asphyxia 59; neonatal jaundice 60 exclusive breast-feeding 61–62; umbilical cord granuloma^63^, cerebral palsy^64^–^65^ and depression^66^.

*Others:* this contains papers on low back pain^67^; musculoskeletal disorder among dentists^68^; and traditional bone setting in Cameroon^69^. There are radiology papers^70^–^72^; community health workers^73^–^75^ and a heterogenous group of papers including dental implants^76^; renal cysts^77^; bearing loss^78^ biometrics^79^; medicinal plants^80^ and a review of NCDs^81^ and thromboembolism^82^. Quite a journey!

*Thank you:* It only remains for us to express our heartfelt thanks and gratitude to the readers, authors, reviewers, collaborative partners, editorial team and all those who have, in one way or another, contributed to the growth and sustenance of *African Health Sciences* since its very humble beginning. That we have now attained an **impact factor of 1.108** up from 0.927 is testimony to the dedication and commitment of individuals and collaborative partners as we strive to uplift publication of science relevant to Africa and the diaspora. Thank you very much and enjoy this June 2022 issue of African Health Sciences. Thank you!
